# The Structure of the Brachial Plexus in Selected Representatives of the Caniformia Suborder

**DOI:** 10.3390/ani12050566

**Published:** 2022-02-23

**Authors:** Arkadiusz Grzeczka, Maciej Zdun

**Affiliations:** Department of Basic and Preclinical Sciences, Nicolaus Copernicus University, 87-100 Toruń, Poland; maciejzdun@umk.pl

**Keywords:** American mink, common raccoon dog, comparative anatomy, European pine marten, neuroanatomy, red fox

## Abstract

**Simple Summary:**

The brachial plexus of animals is an ongoing topic of research interest among the scientific community. Research in this area allows scientists to select model animals for laboratory studies. In this way, the research contributes to the improvement of techniques for performing various procedures, not only on animals but also on humans. The most important information about the brachial plexus is the number of roots that form the plexus, from which roots the individual nerves arise, their course, and the extent of innervation of the individual nerves. In this paper, we have analysed the brachial plexus of five species of animals that commonly live in forests. In the study, we show similarities to the domestic dog but also present the individual anatomical features of the brachial plexus. Our results increase knowledge of the nervous system anatomy of wild animals and provide important information for veterinarians dealing with wild and exotic animal species.

**Abstract:**

Like most structures, the brachial plexus is subject to species variation. Analysing this structure over a wide spectrum of species, we can obtain a complex view of the changes–in a given group of animals. The aim of this study was to describe the brachial plexus anatomy of species from two families of Caniformia. We analysed the brachial plexus structure of five species from two families of Caniformia: Canidae and Mustelidae. The cadavers were obtained from breeders and hunters. All were fixed by being kept in a 10% formaldehyde solution for two weeks. This study allows us to present the similarities as well as the differences between species and families. Our study reveals different trends in the course of the individual nerves and innervations of the thoracic limb. A species-specific feature is the extent of the brachial plexus, as each species has a specific number of ventral branches of the spinal nerves in the brachial plexus. However, a characteristic of the family Mustelidae is the course of the median nerve through the epicondylar foramen. Within the Canidae, two species are characterised by a very long branch for the coracobrachialis muscle. The general conclusion is that the brachial plexus of species belonging to the Caniformia is subject to variation within families and species, as well as individual variation while maintaining a general schematic for the group.

## 1. Introduction

Wild animals of the Caniformia suborder and Carnivora order are a large group with numerous representatives [1]. The most common are representatives of the Mustelidae and Canidae families [2]. These two groups include terrestrial and arboreal animals. Therese animals differ in the development of the muscles of their thoracic limbs [3]. The subject of this study is the brachial plexus (*plexus brachialis*), which provides muscle activation to the thoracic limb. The authors of this study focused on the two most common families of the suborder Caniformia (Mustelidae and Canidae) and attempted to describe them with each other regarding the structure of the brachial plexus and any characteristics of peripheral nerves derived from the brachial plexus. The study was carried out by means of the description of the brachial plexuses of the European pine marten (*Martes martes*), American mink (*Mustela vison*), European badger (*Meles meles*), common raccoon dog (*Nyctereutes procyonoides*), red fox (*Vulpes vulpes*). These animals were obtained from breeders and hunters. The methodology that was used to fix and dissect the plexuses follows the pattern used by other researchers focusing on this topic [4,5]. 

As a result of scientific publications, the structure of the brachial plexus is characteristic of specific groups of animals [4,5,6,7,8,9]. Additionally, canids have characteristic variants of the range of the brachial plexus. The most common range in papers describing the plexuses of Caniformia members is C6-T1 [5,8]. However, some results indicate that the C5 and T2 branches may be involved in plexus formation [8]. There are also species differences in the course of the peripheral nerves of the brachial plexus. In the Canidae species, a connecting branch between the median nerve and the musculocutaneous nerve occurs at the level of the distal humerus [5]. The data obtained from the analysis in this study can be used for comparison with another large group of carnivores, such as felines. Emerging work on this group indicates that the extent of the plexus may be similar to that found in Caniformia [10,11]. Anatomical studies carried out on cats have also shown subtle but significant differences in the nervous system of their forearms [12]. Published papers often lack important information about the brachial plexus. The deficiencies we refer to are the absence of muscle and skin innervation and the lack of detailed presentation of the architecture of the arm innervation [5,8]. This is important because muscle and skin innervation is of great clinical significance. Information about possible muscle paresis or lack of sensation in certain parts of the body is of diagnostic value [13]. We believe that the group to which the domestic dog belongs (*Canis lupus familiaris*), one of the most important species for veterinary medicine, is valuable from a cognitive and comparative point of view. The Caniformia include species of breeding importance and wild species that are commonly found in forests. Therefore, the authors of this paper decided to investigate this group of animals and examine the diverse structure of their brachial plexus and the course of the nerves deriving from it. Despite their commonness, many of these species can be found in zoos, which are reservoirs of genetic material for future generations. For this reason, the knowledge of the innervation of the thoracic limb is of great importance, for example, in the treatment of injuries or inducing local anaesthesia.

The aim of this study was to describe the brachial plexus anatomy of selected Caniformia species.

## 2. Materials and Methods

Adult animals of both sexes of five species of the Carnivora order were included in the study. The following mustelids were analysed: European pine marten (*Martes martes*) (female = 6, male = 2), American mink (*Mustela vison*) (female = 18, male = 12), and European badger (*Meles meles*) (female = 15, male = 10). The following canids were analysed: common raccoon dog (*Nyctereutes procyonoides*) (female = 10, male = 10), red fox (*Vulpes vulpes*) (female = 10, male = 16). As bilateral plexuses were treated individually, the total number of plexuses tested is twice the number of animals. The cadavers of red foxes, European badgers, European pine martens and common raccoon dogs, were obtained through hunting while mink were obtained from a breeding farm. The farm from which the American mink came is under constant veterinary control, and the animals had undergone anti-parasitic prophylaxis and vaccinations against distemper, because of which all the animals on the farm were healthy. In addition, the farm monitors the direction of Aleutian disease. The age and weight of the animals were 18–20 months and 0.950 kg to 1.350 kg respectively. The rest of the species we obtained from hunting. Of the specimens of the species we obtained from hunting, only animals without damage to the thorax and thoracic limb were included in the study. All cadavers were opened via the abdominal and thoracic cavity and then immersed in containers (with a volume of 200l) filled with fixative for two weeks. For the fixation of the cadavers, a 10% solution of buffered formalin was used (Formalin 10%, Chempur, catalogue number—114321735). After fixation, the corpses were rinsed for two days in a pool of water to ensure safe preparation. Safety was also ensured by a ventilation system in the preparation room, which maintained 15 air changes per hour. Preparations began with removing skin from the entire chest and thoracic limbs. When the muscles were exposed, incisions were made on the brachial attachment of the pectoralis muscles, latissimus dorsi muscle and cutaneous trunci muscle. The blood vessels present in this area were then removed. After clearing the nerves of any remaining connective tissue, a 3% hydrogen peroxide solution (Chempur, catalogue no. p.d.a. 118851937) was used. The chemical was applied to the nervous tissue using a cotton swab. The next step to show the extent of innervation was to incise the muscle bellies of the upper arm and forearm. The research was documented by the description of the preparation obtained and by diagrams. A Nikon D3200 camera was used to take the photographs, which were saved in JPG format. GIMP version 2.10.24 and Paint3D were used to process the photos.

## 3. Results

The brachial plexuses of individual species differed in terms of the number of ventral branches (Table 1). The most common plexus in the American mink consisted of C6-T2 (plexus (*p*) = 48). The brachial plexus of the European pine marten, which is closely related to the American mink, consisted of C6-T1 only (*p* = 16). The brachial plexus of the third species of the Mustelidae family, i.e., the European badger, covered the C6 to T2 in 25 cases, but the C6-T1 range was also frequent (*p* = 20), whereas the C5-T1 range was the least frequent (*p* = 5). The Canidae family was also characterised by the presence of the T2 branch in the brachial plexus (C6-T2) (Table 1). Additionally, the brachial plexus of the common raccoon dog was most often connected with the C5 branch (*p* = 5). This branch was also observed in the plexus of the red fox (*p* = 4).

The following nerves were found in each of the animals under study: radial (*n. radialis*), median (*n. medianus*), ulnar (*n. ulnaris*), musculocutaneous (*n. musculocutaneus*), axillary (*n. axillaris*), thoracodorsal (*n. thoracodorsalis*), suprascapular (*n. suprascapularis*), subscapular (*nn. subscapulares*), long thoracic (*n. thoracicus longus*), lateral thoracic (*n. thoracicus lateralis*), cranial pectoral (*nn. pectorales craniales*), caudal pectoral (*nn. pectorales caudales*), and brachiocephalic nerve (*n. brachiocephalicus*). The branches from which these nerves derived were also examined in each species (Table 2).

In each of the animal species under analysis, except the red fox, the plexuses formed nerve trunks as a result of the connection of individual ventral branches of the nerve roots. The nerves of the red fox did not form trunks. In the other species, trunks were formed from various branches of the ventral nerve roots. The cranial trunk consisted only of the C6 branch, except for the trunk of the European badger, where 5 of the plexuses also consisted of the C5 branch. The middle trunk of all species consisted of the C7 branch. The brachial plexus of the American mink and European pine marten was characterised by a relatively greater distance between the cranial and caudal trunks due to the fact that the bodies of these two species were significantly elongated. The caudal trunk always consisted of C8 and T1. Additionally, it sometimes included the T2 branch. In the further part of the plexus, in each of the animals that had the trunks, there was a split into two divisions. The way the divisions were intertwined and the formation of nerves enabled the identification of differences between the species. In this area of the plexus, the biggest differences were found in the axillary and radial nerve systems. There were differences in the branch connecting these two nerves. The branch was very short in the European badger, and the nerves seemed to be connected at some point. This branch was distinct in the other species, but there were differences in where it joined the other nerves. The location of the ends of this branch in the mink meant that it was positioned obliquely, which was similar to the location in the red fox. However, it was perpendicular to both nerves in the European pine marten and common raccoon dog.

In the peripheral part of these nerves, there were differences in the number of muscle branches of the axillary nerve before it entered the groove of the subscapularis muscle. The American mink and European badger had two separate branches for the subscapularis muscle and the teres major muscle. The European pine marten and common raccoon dog had one branch, which split into two branches for the abovementioned nerves. The red fox also had one branch, which split for these muscles, but there were two branches for the subscapularis muscle and one for the teres major muscle.

**Figure 1:**
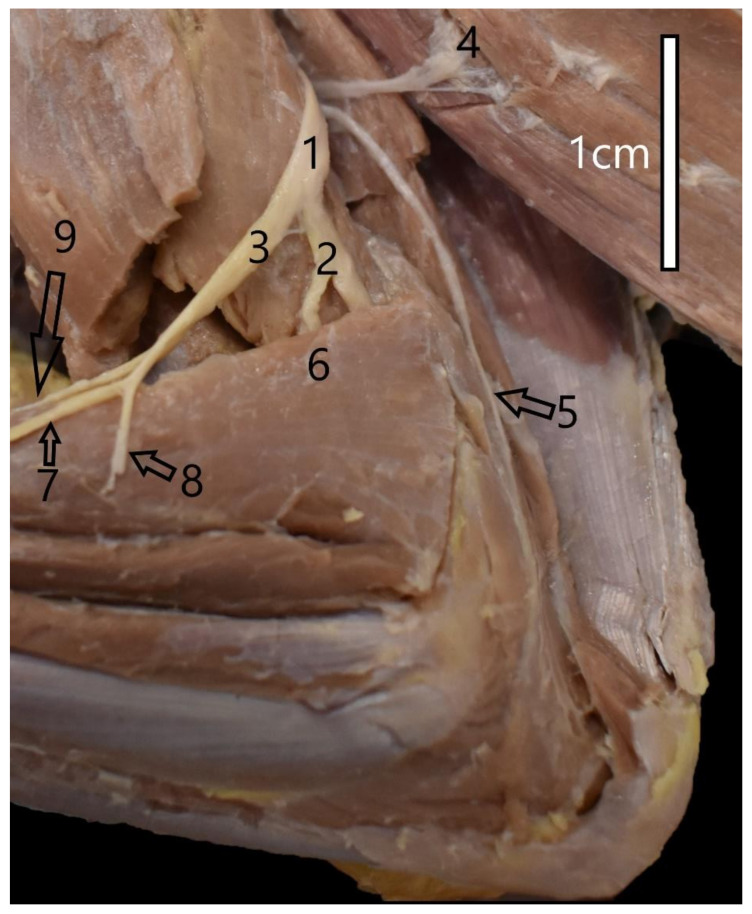
Innervation of the left arm of the common raccoon dog (*Nyctereutes procyonoides*). 1—Radial nerve; 2—Deep branch of the radial nerve; 3—Superficial branch of the radial nerve; 4—Branch of the radial nerve to lateral head of the triceps muscle of the arm; 5—Branch of the radial nerve to anconeus muscle; 6—Branch of the radial nerve to brachioradialis muscle; 7—Lateral branch of the superficial branch; 8—Lateral antebrachial cutaneous nerve; 9—Medial branch of the superficial branch.

The other of the abovementioned nerves, i.e., the radial nerve, was the biggest in the brachial plexus. It innervated the most muscles. It was formed from the C7-T1 branches. At the forearm level, it innervated the tensor muscle of the antebrachial fascia, the triceps brachii muscle, and the ulnar muscle. In all the species under analysis, the radial nerve passed to the lateral surface of the arm and divided into the deep and superficial branches (Figure 1). At the shoulder level, it is also divided into the cutaneous branch in the form of the lateral brachial cutaneous nerve (*n. cutaneus brachii lateralis caudalis*). The deep branch innervated the dorsolateral muscles of the forearm. The superficial branch is divided into its lateral and medial parts and innervated the palm and the skin on the lateral side of the forearm via the lateral antebrachial cutaneous nerve (*n. cutaneus antebrachii lateralis*). The lateral branch is divided into the common dorsal digital nerve I and the abaxial dorsal digital nerve I. The medial branch is divided into the common dorsal digital nerves II, III, and IV. There were differences in the quality of the palmar nerves of digit I. The branch for digit I was much thinner than the nerves of the other digits in the animals of the Canidae family. These nerves were of equal quality in the mustelids.

When the musculocutaneous and median nerves merged, the fibres interlaced in a species- or family-specific manner. The communicating branch appeared in two places: in the axilla and at the level of the elbow joint. This branch was found in the axilla of all animals except the red fox. This branch was regularly found at the level of the elbow in the red fox. Two branches could be found in the European badger. However, both were located in the axilla and differed significantly in diameter and length.

**Figure 2:**
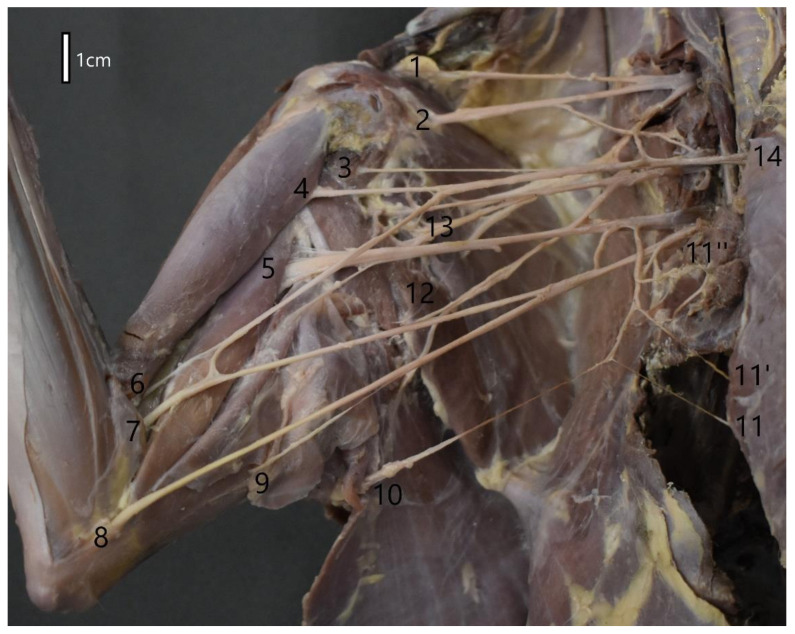
Structure of the right brachial plexus of the red fox (*Vulpes vulpes*). 1—Brachiocephalic nerve; 2—Suprascapular nerve; 3—Branch of the musculocutaneus nerve to coracobrachialis muscle; 4—Branch of the musculocutaneus nerve to biceps muscle of the arm; 5—Radial nerve; 6—Musculocutaneus nerve; 7—Medial nerve; 8—Ulnar nerve; 9—Part of the caudal antebrachial cutaneus nerve; 10—Lateral thoracic nerve; 11,11′,11′′—Caudal pectoral nerves; 12—Thoracodorsal nerve; 13—Axillary nerve; 14—Cranial pectoral nerve.

**Figure 3:**
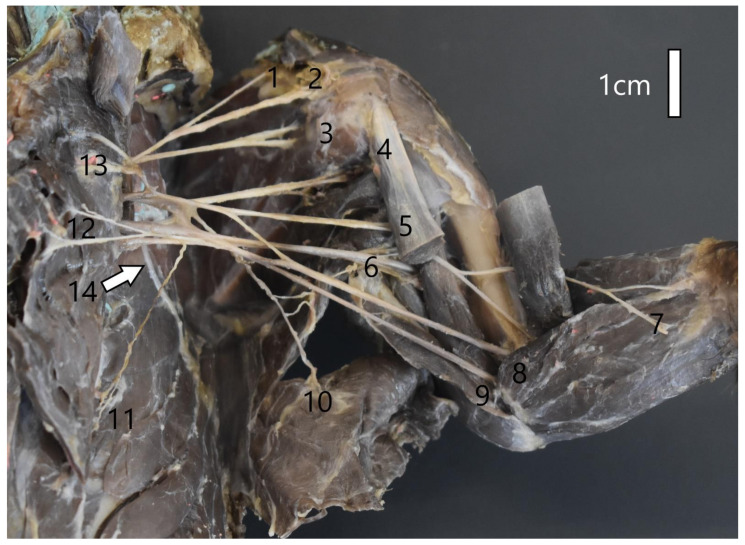
Structure of the left brachial plexus of the American mink (*Mustela vison*). 1—Brachiocephalic nerve; 2—Suprascapular nerve; 3,—Subscapular nerve; 4—Axillar nerve; 5—Musculocutaneus nerve; 6—Radial nerve; 7—Medial antebrachial cutaneous nerve; 8—Median nerve; 9—Ulnar nerve; 10—Thoracodorsal nerve; 11—Lateral thoracic nerve; 12—Caudal pectoral nerve; 13—Cranial pectoral nerve; 14—Long thoracic nerve.

The muscle branches of the musculocutaneous nerve innervate the coracobrachialis, biceps, and brachial muscles. The distribution of the branches innervating these muscles is family-specific. The branch for the coracobrachialis muscle and biceps, which appeared only next to the muscle itself, was characteristic of the Mustelidae family and the common raccoon dog. It was one branch that split into these two muscles. In the red fox, the rami for the coracobrachialis muscle and biceps were separate and branched much earlier (Figure 2). The ramus for the brachialis muscle branched at the level of the elbow joint. In the red fox, it branched before the ramus connecting with the median nerve. In each case, the musculocutaneous nerve ended in the skin at the forearm level as the medial antebrachial cutaneous nerve (*n. cutenaus antebrachiali medialis*). The median nerve did not branch into rami at the shoulder level but innervated various antebrachial muscles and the palm (Table 2). However, in the plexuses of the animals of the Mustelidae family, the median nerve first passed through the supracondylar foramen together with the brachial artery before it crossed the elbow joint (Figure 3). After crossing the line of the elbow joint, the median nerve followed the same course in all the species. At the forearm level, it is divided into muscle branches, the main stem, and the anterior interosseous nerve, innervating the periosteum. Having passed through the wrist level, the median nerve branched into three nerves: common palmar digital nerve I, common palmar digital nerve II, and common palmar digital nerve III.

**Figure 4:**
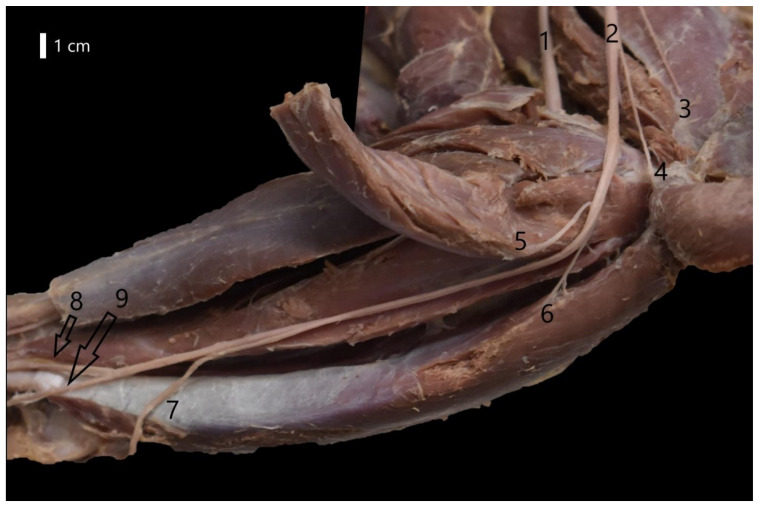
Course of the ulnar nerve at the forearm level in the European badger (Meles meles). 1—Median nerve; 2—Ulnar nerve; 3—Part of the caudal antebrachial cutaneus nerve; 4—Brach of the ulnar nerve to ulnar head of the flexor carpi ulnaris muscle; 5—Branch of the ulnar nerve to humeral head of the flexor digitorum profundus muscle; 6—Branch of the ulnar nerve to humeral head of the flexor carpi ulnaris muscle; 7—Dorsal branch of the ulnar nerve; 8—Superficial branch of the palmar branch of the ulnar nerve; 9—Deep branch of the palmar branch of the ulnar nerve.

The ulnar nerve branched into a cutaneous ramus, i.e., the caudal antebrachial cutaneous nerve (*n. cutaneus antebrachii caudalis*) and articular branches. Then it went towards the ulnar process and passed near the medial epicondyle. At the forearm level, together with the median nerve, it innervated the medial abdominal muscles. Before the wrist, it branched into the dorsal ramus, which innervated digit V (Figure 4). At the wrist level, it divided into the superficial branch and the deep branch. The deep branch ran towards the metacarpal bones, whereas the superficial branch ran towards the distal ends of the digits, forming the common palmar nerves. At the level of the digits, the proper palmar digital nerves IV and V and the abaxial digital nerve V were formed. The course of the ulnar nerve was consistent with this pattern in all the species.

**Figure 5:**
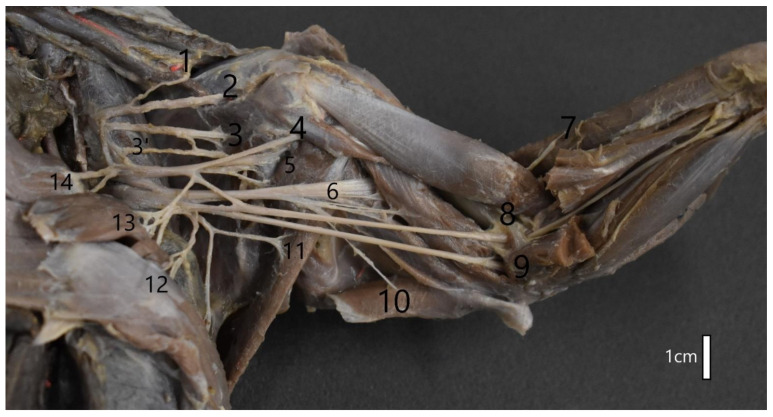
Structure of the left brachial plexus of the European pine marten (*Martes martes*). 1—Brachiocephalic nerve; 2—Suprascapular nerve; 3,3′—Subscapular nerve; 4—Musculocutaneus nerve; 5—Axillar nerve; 6—Radial nerve; 7—Medial antebrachial cutaneous nerve; 8—Median nerve; 9—Ulnar nerve; 10—Branch to tensor fasciae antebrachii; 11—Thoracodorsal nerve; 12—Lateral thoracic nerve; 13—Caudal pectoral nerve; 14—Cranial pectoral nerve.

The nerves of the pectoral muscles differed in terms of the number of fibres innervating these muscles and the roots from which the fibres forming the appropriate pectoral nerve derived. The cranial pectoral nerves had four different forms, the most common of which was two separate cranial pectoral nerves, where one was formed only from C7 and the other from C7-C8. This pattern was observed in the common raccoon dog and European badger. The cranial pectoral nerves of the American mink were formed as a result of the division of one thicker branch (C7). There was a similar pattern in the red fox, but it had an additional ramulus leading to another part of the superficial pectoral muscle, which branched before the division of the main ramus (C6-C7). The cranial pectoral nerves of the European pine marten had two branches formed from C7 (Figure 5). There were three forms of the caudal pectoral nerves: from the C7-T1 rami, from the C8-T1 rami, or from the C8-T2 rami (Table 2). The caudal pectoral nerves of the European pine marten shared the trunk with the lateral pectoral nerve. The trunk branched into the caudal pectoral nerves and then extended towards the pectoral cutaneous nerve. The long thoracic nerve and the thoracodorsal nerve were the last nerves innervating the pectoral muscles. The long thoracic nerve was formed mainly from the C7 branch (badger, fox), but sometimes there were additional branches from C6 (raccoon dog) or C8 (mink, marten). The long thoracic nerve innervated the serratus ventralis thoracis muscle. The thoracodorsal nerve was mainly formed from C7-C8 (marten, badger, fox). However, it was also formed from C7-T1 in the mink and raccoon dog. 

Additionally, this nerve was responsible for the innervation of one muscle, the latissimus dorsi.

The brachiocephalic nerve was formed mainly by the C6-C7 branches. However, if the C5 branch joined the plexus, it also formed this nerve. In each of the species, the brachiocephalic nerve innervated the cleidobrachialis muscle and, with the help of the cutaneous branch, innervated the skin on the cranial side of the brachial joint. The suprascapular nerve, which was formed by similar branches (C6-C7) in all the species, innervated the scapular muscles, i.e., the supraspinatus and infraspinatus.

The subscapular nerves, which also innervated the scapular muscles, occurred in the form of two nerves. The only exceptions were the red fox and American mink, where the common part formed for the subscapular nerves divided only after some time.

## 4. Discussion

In our study, we showed that the plexus of Caniformia representatives may include C5-T2 roots. A study on the Pampas fox (*Lycalopex gymnocercus*) showed that the ranges C6-T1 and C6-T2 is also characteristic of this group [5]. There were similar results of the analysis of the nerve roots in the red fox (*Vulpes vulpes*) (C6-T2), as well as in the crab-eating fox (*Cerdocyon thous*) (C6-T1), South American fur seal (*Arctocephalus australis*) (C6-T1), beech marten (*Martes foina*) (C6-T1), and short-eared dog (*Atelocynus microtis*) [8,14,15,16,17]. A study on the South American coati (*Nasua nasua*) showed that the brachial plexus of animals of the Caniformia suborder does not always have to include the T1 branch [18]. In this study, the frequency of the C5-C8 variant was 40%, which was equal to the frequency of the C6-T1 variant in this species. This means that T1 did not contribute to the formation of the plexus in 40% of the animals under study. It is also noteworthy that, in the domestic dog, which is the best-known representative of canines, the brachial plexus is reported to range between C6 and T2. However, there may be additional connections of the C5 branch, or the T2 branch may be absent [19,20]. The data on the number of ventral rami of the cervical and pectoral nerves presented in this study differ from some orders of mammals. The authors of the study conducted on animals of the Rodentia order, noted a wider range of branches included in the brachial plexus. For example, the range of the brachial plexus in representatives of the Muroidea superfamily, e.g., Mongolian gerbil (*Meriones unguiculatus*), is C4-T1 [4]. However, there was one case each where it was C6-T3, the Spix’s yellow-toothed cavy (*Galea spixi*), and C5-T1—the Gambian pouched rat (*Cricetomys gambianus*) [21,22]. In even-toed ungulates this range is similar to the Caniformia, e.g., the okapi (*Okapia johnstoni*) is C6-T1, and the grey brocket (*Mazama gouazoubira*) is C6-T1 [6,23,24]. The comparison with the Feliformia suborder, which is another large group of wild predators, showed that some species did not have connections with C5 and T2, e.g., Van cats [11]. One study was performed on a domestic cat and it was observed that in some cases T2 was involved in the formation of the Feliformia brachial plexus [25]. The presence of the supracondylar foramen in the path of the median nerve is a common feature of the Feliformia and Mustelidae family members [26,27,28]. The presence of this foramen is also characteristic of marsupials, dolphins, koalas, and kangaroos [29]. It can also be found in humans, primates, some rodents, and numerous reptiles [30,31,32,33]. According to some authors, the supracondylar foramen stabilises the median nerve and is equivalent to the retinaculum, which occurs, for example, at the wrist level [29]. In our study, the median nerve passed through the supracondylar foramen, together with the brachial artery. In wild felids, the median nerve was accompanied by the brachial artery and the brachial vein when passing through the supracondylar foramen of the humerus. However, in domestic cats, the vein separated at this site [12,34,35]. In our study, the Canidae did not have the supracondylar foramen. This observation is in line with Landry’s theory, which explains the absence of the supracondylar foramen from animals of the Canidae family by the fact that their axilla is relatively short in comparison with the axilla in the Mustelidae. Therefore, the skin is much tenser at the site where the median nerve passes, so there is no need for any bone element to support the median nerve [29].

Except for the brachiocephalic nerve, all the nerves described in this manuscript are listed in the Nomina Anatomica Veterinaria (NAV) [36]. Many authors also classified this nerve as part of the brachial plexus due to its origin and functions [9,37]. They named it the brachiocephalic nerve. It innervates part of the brachiocephalic muscle, i.e., the cleidobrachialis. The author of the study describing the brachial plexus of the beech marten distinguished the subclavian nerve (*n. subclavius*), which was characterised by an identical supply area and the same origin [15]. The subclavian nerve was also listed in some studies on the brachial plexus of rodents [38,39]. However, it was not mentioned in studies on some felids and rodents [4,21]. These changes in nomenclature may have resulted from significant differences in the occurrence of collarbones among mammals. It can be found in various species of rodents, but it occurs in a residual form in the Carnivora [40,41].

The area innervated by individual nerves in this group of animals is essentially consistent. However, as can be seen in Table 2, the ulnar nerve of the European pine marten also innervates the superficial flexor muscle. The author of a study on the beech marten, which is a closely related species to the European pine marten described in our study, did not provide details on the muscles innervated by the ulnar nerve [15]. It is also noteworthy that the innervation of the distal part of the humeral head of the deep digital flexor is specific to the Caniformia, but was not reported by the authors of studies on felids [11]. The innervation of the brachioradialis is also characteristic because it is a very thin muscle that can often be removed with the skin. It is even absent from some species or is residual [42,43]. The innervation of the superficial digital flexor muscle by the median nerve is also characteristic of carnivores [9,11,44]. The similarities demonstrated in our study suggest that it is possible to use the techniques of surgery and anaesthesia applied to the domestic dog [45,46,47]. The knowledge of the anatomy of nerves and muscle innervation is used in clinical diagnostics— for example, to differentiate injuries in the brachial plexus and around the periphery of the thoracic limb [48,49,50,51,52].

Mustelids use their pectoral limbs to move around, dig burrows, climb trees, and hold their prey. On the other hand, except for the fox, the Canidae described in our study rarely dig burrows. Instead, they use the habitats left by other species of similar size, as the raccoon dog does. This diversity causes differences in the activity of areas in the sensorimotor cortex. This fact was confirmed by research that showed that specific areas of the cerebral cortex are activated depending on the task performed [53]. The faster and more accurate the tasks, the more strongly the centres under study were activated. This suggests that animals’ behavioural activities, e.g., European badgers digging burrows, may result in greater development of the brain areas responsible for these activities, as compared with the European pine marten, which uses abandoned hollows, burrows, and crevices in rocks. Skillful performance of individual activities also affects the morphological image of the motor cortex [54]. Therefore, the complexity and quality of palm innervation may suggest differences in the structure of the motor cortex that occur within the medial sulcus and medial gyrus in humans, as observed by the authors of the study cited above. These differences may also occur in the motor areas of the cingulate gyrus and sensorimotor areas of the encephalon [53,54]. Our research showed that the biggest differences in the palm innervation between the Canidae and Mustelidae families were observed in digit I. The described differences suggesting the stronger innervation of digit I in the Mustelidae family may be related to the fact that members of the Canidae family do not use their digit I to move around, so its innervation is not as important as in the Mustelidae. This might be why the centres responsible for the performance of manual tasks with the pectoral limb are more developed in the Mustelidae. The investigation of all evolutionary and environmental factors influencing the variability of the central and peripheral nervous systems in other species than the model ones makes it possible to understand some changes occurring in the nervous system and provides new factors for the classification of animals.

## 5. Conclusions

In conclusion, the overall structure of the brachial plexus appears to be consistent in all the species we studied. The roots that could be part of the plexus of the species studied are C5, C6, C7, C8, T1 and T2. The range of plexus that we have demonstrated in individuals correlates with the number of roots indicated in other species of Caniformia. However, there are species and individual differences. Variations included features, such as range of plexus, nerve formation by different nerve roots, connections between nerves, and differences in the innervation of the thoracic limb muscles. This information is important for researchers conducting macroanatomical comparative studies. The results of our study may also be useful for veterinarians dealing with wild species of animals related to dogs.

## Figures and Tables

**Table 1:** The range of the brachial plexus in species examined (the numbers indicated refer to the number of plexuses (*p*) examined in each species).

Animals	Brachial Plexus Range	Number of Plexuses on Each Side	
		L	R
American mink (*Mustella vision*)	C6-T1	4	8
	C6-T2	26	22
European pine marten *Martes martes*)	C6-T1	8	8
European badger (*Meles meles*)	C5-T1	0	5
	C6-T1	15	5
	C6-T2	10	15
Red fox (*Vulpes vulpes*)	C5-T1	4	0
	C6-T1	14	18
	C6-T2	8	8
Common raccoon dog (*Nyctereutes procyonoides*)	C5-T1	0	5
	C6-T1	5	5
	C6-T2	15	10

**Table 2:** Origin of brachial plexus nerves in the species studied and their innervation range.

Nerves	American Mink	European Pine Marten	European Badger	Red Fox	Common Raccoon Dog	Innervated Muscle
Radial	C7-T1 C7-T2	C1-T1	C7-T1 C7-T2	C7-T1 C7-T2	C7-T1 C7-T2	Tensor fasciae antebrachii, triceps of the arm, anconeus, extensors of wrist and fingers, brachioradialis
Axillar	C7-C8	C6-C8	C7-C8	C6-C7	C6-C8	Subscapularis, teres major, teres minor and deltoideus
Median	C7-T1 C7-T2	C7-T1	C8-T1	C8-T1	C7-T1 C8-T1	Pronator teres, flexor carpi radialis, flexor digitorum profundus (radial and humeral heads), pronator quadratus, flexor digitorum superficialis
Ulnar	C8-T1 C8-T2	C8-T1	C8-T1 C8-T2	C8-T1 C8-T2	C8-T1 C8-T2	Flexor carpi ulnaris and flexor digitorum profundus (ulnar and humeral heads), flexor digitorum superficialis (in the European pine marten)
Musculocutaneus	C6-C7	C6-C7	C6-C7	C6-C7	C6-C7	Coracobrachialis, biceps brachii and brachialis.
Suprascapular	C6-C7	C6-C7	C5-C7 C6-C7	C5-C7 C6-C7	C5-C7 C6-C7	Supraspinatus and infraspinatus
Subscapular	C6-C7	C6-C8	C6-C7	C6-C7	C6-C8	Subscapularis
Cranial pectoral	C7	C7	C7 C7-C8	C6-C7	C7 C7-C8	Pectorales superficiales
Caudal pectoral	C7-T1	C7-T1	C8-T1 C8-T2	C8-T1 C8-T2	C8-T1 C8-T2	Pectorales profundus
Longus thoracic	C7-C8	C7-C8	C7	C7	C6-C7	Serratus ventralis
Lateral thoracic	C8-T2	C8-T1	C8-T1 C8-T2	T1-T2	C8-T1 C8-T2	Cutaneous trunci and pectorales profundus (in the European pine marten and red fox)
Thoracodorsal	C7-T1	C7-C8	C7-C8	C7-C8	C7-T1	Latissimus dorsi
Brachiocephalic	C6	C6	C5-C6	C6	C5-C6	Cleidobrachialis
